# Ytterbium oxide nanofibers: fabrication and characterization for energy applications

**DOI:** 10.55730/1300-0527.3472

**Published:** 2022-08-15

**Authors:** Adem SARILMAZ

**Affiliations:** Department of Metallurgical and Materials Engineering, Faculty of Engineering, Karamanoğlu Mehmetbey University, Karaman, Turkey

**Keywords:** Yb_2_O_3_, ytterbium oxide, nanofiber, electrospinning, band-gap calculation

## Abstract

Metal oxide nanomaterials are widely used in many applications of renewable energy. Ytterbium oxide (Yb_2_O_3_) also attracts attention due to its similar structure to ZnO (~3,26 eV) and TiO_2_ (~3,36 eV) in terms of bandgap energy. In this study, Yb_2_O_3_ belonging to the lanthanide oxide family was produced as nanofiber forms by using the electrospinning technique, which allows for large-scale production, for the first time. The morphological, structural, optical, and phase properties of the produced nanofibers were investigated via XRD, SEM-Mapping, TEM, FTIR, UV-Vis, and XPS characterization techniques. As a result of these analyses, it was determined that the Yb_2_O_3_ nanofibers with a diameter of 125 ± 15 nm have a cubic crystal structure and a bandgap of 3.32 eV. The results of this study have shown that the Yb_2_O_3_ nanofibers are capable of performing performance evaluations in many different energy conversion applications and others.

## 1. Introduction

The energy needs of humanity have increased in parallel with the growth of the earth’s population. In the last century, the scientific world has focused on identifying environmentally friendly new alternative energy sources due to reasons such as limited fossil fuel sources and damage of these fuels to nature. When the developments in this context are examined, it is seen that the performed studies are focused on energy production from natural and renewable sources such as the sun, wind, and water. As it is known, metal oxide materials are extensively used in energy production from renewable energy sources. Literature review shows that ytterbium oxide and ytterbium-doped metal oxide nanomaterials belonging to the lanthanide oxide family are interesting materials within the scope of these studies [1–6]. For example, it has been reported in many studies that ytterbium-based or ytterbium-doped oxides increase photoactivity in hydrogen evolution reactions (HER) [3, 5, 7–9]. In addition to HER studies, these materials have been used as photoanode in dye-sensitized solar cells (DSSCs) and as upconverted layers in thin-film solar cells. It has been reported in the DSSC study that when ytterbium-based materials are used together with photoanodes such as TiO_2_ and ZnO, they facilitate charge transport and increase the lifetime of photoexcited electrons [1, 6]. On the other hand, they help to absorb shorter wavelengths and thus increase the power conversion efficiency in the thin-film solar cell [10].

As can be understood from the previous studies, nanostructured Yb_2_O_3_ can be produced in different morphologies with various synthesis methods such as hydrothermal [11–13], solvothermal [14], precipitation [15, 16], and electrospinning [17]. Among the synthesis methods, electrospinning which is an environmentally friendly, low-cost, and simple production method is a common production technique used in nanomaterials production. This production technique generally consists of four main components: the high voltage power supply, syringe with a metal needle, syringe pump, and sample collector. The voltage range, the distance between the syringe with the collector, and feed rate are the main parameters for nanofiber production. The changes in these parameters allow the morphological properties of the produced nanofibers to be controlled. Therefore, electrospinning facilitates the production of nanofibers with porous, high aspect ratio, and large surface area. As it is known, the morphological properties of the produced materials are an important parameter for energy applications. These properties of nanofibers contribute to increasing efficiency in energy applications [18, 19]. As mentioned at the beginning of the paragraph, the synthesis routes of nanostructured Yb_2_O_3_ are limited, and it was reported that Yb_2_O_3_ was produced by electrospinning in only one study. As seen from TEM and SEM images of Yb_2_O_3_ produced within the scope of this study, the obtained nanomaterials formed as irregular nanoparticles rather than nanofiber [20]. In other nanofiber studies, ytterbium has been used as a dopant material in metal oxide nanofibers [21–24].

It has been understood from the literature review that the synthesis studies of Yb_2_O_3_ nanostructures are limited, and there is no report on the production of their well-crystallized nanofiber. The production of homogeneous and highly crystalline Yb_2_O_3_ nanofibers by electrospinning has been reported for the first time in this study. Moreover, a detailed bandgap analysis of Yb_2_O_3_ nanofiber has also been carried out. These band gap studies show that the obtained highly crystalline nanofibers can be an alternative to ZnO and TiO_2_, which are used extensively in energy applications.

## 2. Materials and methods

### 2.1. Materials

Ytterbium(III) acetate hydrate (YbAc_3_, 99.95%) and Polyacrylonitrile (PAN, Mw = 150 000 g mol^−1^) were provided by Aldrich. *N, N*-Dimethylformamide (DMF) was supplied from Isolab.

### 2.2. Synthesis procedure of Yb_2_O_3_ nanofibers

Yb_2_O_3_ nanofibers were produced by the electrospinning technique in parallel with previous studies [25, 26]. Typically, 1 mmol YbAc_3_ was dissolved in 5 mL DMF and then 400 mg PAN (8% w/v) was added to the solution. This mixture was stirred overnight to obtain a homogeneous solution. The homogenized solution was then placed in the syringe with a 21-gauge needle and the feed rate of the syringe pump was set as 1 mL/h.

**Figure 1:**
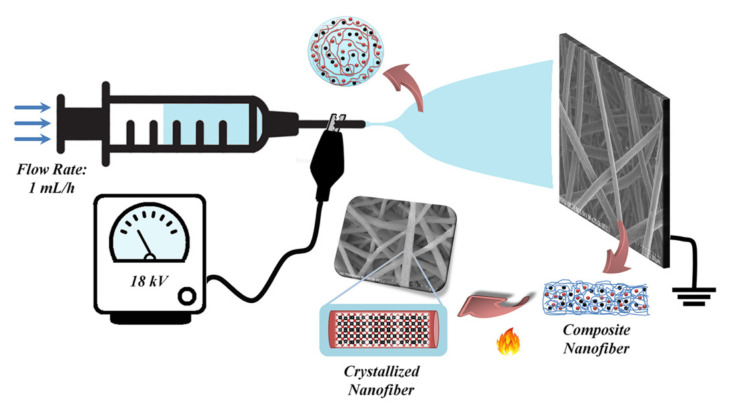
Schematic illustration of the production process of Yb_2_O_3_ nanofibers.

The sample collector was fixed vertically opposite the syringe pump and its distance from the needle was adjusted to 15 cm. After the solution is prepared and the system requirements are set as described, voltage with 18 kV was applied to the metallic needle for the required electric field causing the formation of nanofibers and the sample collector was grounded. Thus, YbAc_3_+PAN nanofibers were collected on aluminum foils on the sample collector and these nanofibers were calcined at 500 °C for 1 h. In this way, the polymer used as a template and other volatile substances in the environment were removed and Yb_2_O_3_ nanofibers were obtained. The process steps of this technique are shown schematically in Figure 1.

## 3. Results and discussions

**Figure 2:**
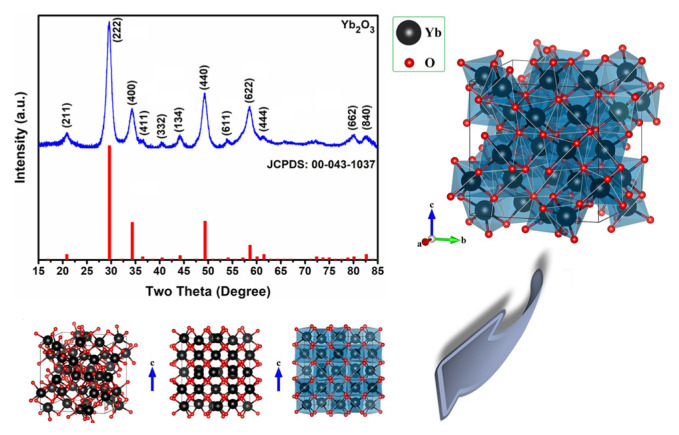
XRD pattern and 3D-crystal structures of the Yb_2_O_3_ nanofibers (these crystal structures were plotted with Vesta software).

First of all, powder X-ray diffraction (XRD) was employed as a bulk analysis technique to confirm the phase structure and purity of the Yb_2_O_3_ compound. Figure 2 demonstrates the XRD pattern of the Yb_2_O_3_ nanofibers. In this structure, the distinctive diffraction signals indicate that Yb_2_O_3_ has a cubic structure (JCPDS card no. 043–1037) with space group Ia-3. The unit cell of this structure consists of 44 polyhedra centered on Yb, and in this structure, each ytterbium atom bonds with 6 oxygen atoms (Figure 2).

The XRD results show that all diffraction peaks are smooth and intense. At the same time, the absence of peaks belonging to another phase structure confirmed the purity of the Yb_2_O_3_ nanofibers. Moreover, the crystallite size distributions were calculated from the XRD pattern by the modified Scherrer’s equation and Williamson–Hall method, and the graphs of these measurements were given in the supplementary part (Figures S1 and S2). The crystallite size of the particles that make up the nanofibers was estimated as 6.53 nm and 6.74 nm by modified Scherrer’s equation and Williamson–Hall method, respectively.

**Figure 3:**
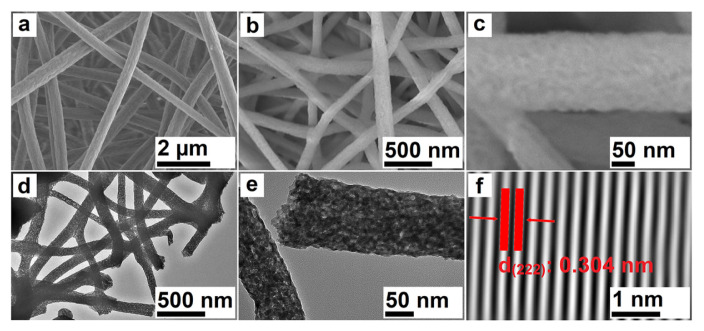
SEM (a–c), TEM (d,e), and HR-TEM (f) characterization results of YbAc_3_+PAN (a) and Yb_2_O_3_ (b–f) nanofibers (particle size and fiber diameter were measured by using ImageJ).

Morphological, crystalline, and compositional properties of the nanofibers were studied by SEM, TEM, elemental mapping, and EDX characterization methods. SEM images of nanofibers pre- and postcalcination process were given in Figures 3a–3c, respectively. These results show that nanofibers have homogeneous size distribution. Moreover, it was determined that the diameters of precalcination nanofibers were 450 nm, and after the heat treatment, the diameters of the crystallized nanofibers decreased to 125 nm due to the loss of organic components. As a result of the performed measurements, the diameter distribution graphs of composite and crystalized nanofibers are given in Figures S3 and S4.

The morphological and crystal properties of nanofibers were detailed by TEM and HR-TEM analyses and the obtained results are given in Figures 3d–3f. It can be seen from the TEM images, the produced nanofibers have homogenous size distribution and this result is also consistent with the SEM results. Figure 3e clearly shows that the nanofibers consisted of Yb_2_O_3_ nanoparticles with about 9 **±** 2 nm. Additionally, this measurement is compatible with crystallite size calculation from the XRD pattern by the modified Scherrer’s equation and Williamson–Hall method. These nanoparticles, which are in contact with each other, were sintered with the effect of heat treatment and thus the nanofiber form was preserved.

The crystallinity of nanofibers was also investigated by the HR-TEM image given in Figure 3f. The perfectly and regular arrangement of the atoms indicates that the fabricated nanofibers have high crystallinity. Moreover, the performed calculations from the XRD pattern show that nanofibers have an 82.75% crystallinity percentage, and this estimate is compatible with TEM results. The process steps belonging to the determination of crystallinity percentage were explained in the supplementary part.

**Figure 4:**
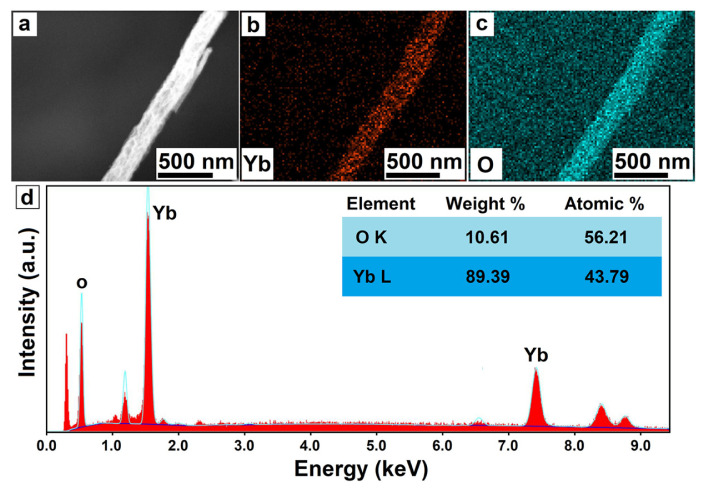
SEM image where EDX and elemental mapping analyses were carried out (a). elemental mapping (b,c), and EDX (d) results of Yb_2_O_3_ nanofibers.

Furthermore, the interplanar spacing was calculated as 3.04 Å, and this value corresponds to the (222) crystallographic plane. In the XRD diffraction pattern, the most intense peak at 29.5° corresponds with the (222) plane, which indicates that the nanoparticles contained in the nanofibers are largely arranged in the (222) plane. Therefore, the obtained results from HR-TEM are compatible with XRD analysis. The elemental distribution and composition of the nanofibers were studied by elemental mapping and EDX analysis methods. Figures 4a–4c reveal that Yb and O elements were homogeneously distributed in the Yb_2_O_3_ nanofibers. Moreover, the chemical composition was calculated as Yb_2.19_O_2.81_ from the EDX spectrum (Figure 4d), and these values are quite close to theoretical ratios.

**Figure 5:**
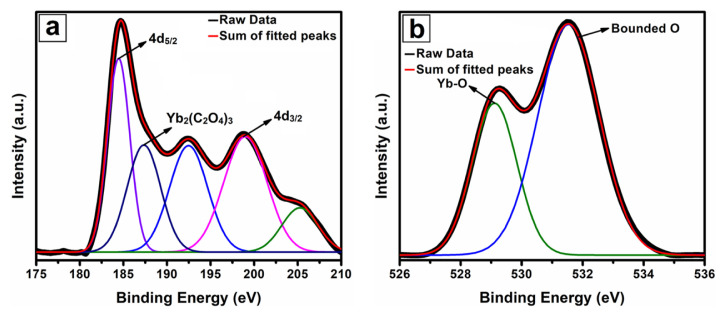
The high resolution XPS spectrum of Yb 4d (a) and O1s (b).

The chemical valance state of the nanofibers was analyzed by the XPS spectra. The high-resolution XPS spectra of Yb 4d and O 1s are given in Figure 5. Yb 4d signals were fitted with five singlet peaks by Gaussian profile (Figure 5a). The peaks centered at 184.4 and 198.8 eV were associated with spin-orbit-split 4d_5/2_ and 4d_3/2_, respectively, and satellite peaks were also detected at 192.4 and 205.3 eV. The energy difference of spin-orbit-split was found to be 14.4 eV as reported in the literature, and it was attributed to the 3+ valance state of Yb [27, 28]. In addition, the peak at 187.3 eV was correlated to the presence of ytterbium oxalate [29]. It was commented that ytterbium oxalate may have occurred from the reaction of YbAc_3_ with organic molecules in the polymer due to the effect of applied temperature during the oxidation of nanofibers. The O 1s high-resolution spectra fitted to two singlet peaks was displayed in Figure 5b. As a result of performed fitting, it was determined that the peak centered at 529.1 was related to O^2−^ ions in the Yb_2_O_3_ crystal structure, while the peaks at 531.5 eV were caused by the hydroxyl group and oxygen vacancies [30].

**Figure 6:**
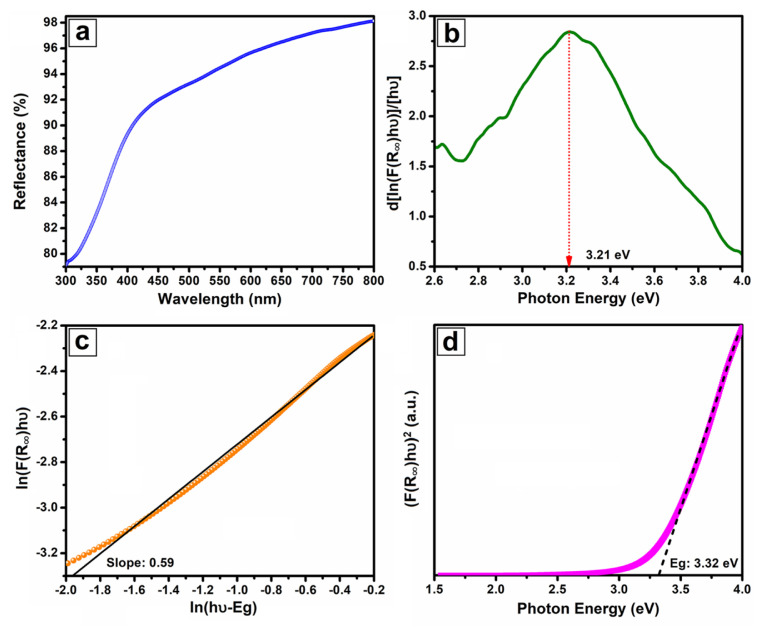
Diffuse reflectance (a), d[ln(F(R_∞_)hυ)]/[ hυ] vs. photon energy graph (b), ln(F(R_∞_)hυ) vs. ln(hυ-E_g_) graph (c), and band-gap energy diagram (d) of Yb_2_O_3_ nanofibers.

The optical properties of nanofibers were investigated by diffuse reflectance spectra and their result is given in Figure 6a. According to this graph, nanofibers have weak reflectivity in the ultraviolet region, while this value increases towards the visible part and reaches a maximum in the near-infrared region. The obtained data from diffuse reflectance spectroscopy were used to calculate the band-gap and determine its type. Firstly, the absorption (F(R_∞_)) was calculated from the Kubelka–Munk equation given in Equation 1.


Equation 1 $$\text{F}({\text{R}}_{\infty })=\frac{{\left(1-R\right)}^{2}}{\left(2R\right)}$$

where *F(R_∞_)* is absorption and *R* is reflection.

The absorption value was used to estimate the approximately band-gap (3.21 eV), which is determined by the graph of d[ln(F(R_∞_)hυ)]/d[hυ]-hυ (Figure 6b). The m exponent was assigned from the slope of the ln(F(R_∞_)hυ)-ln(hυ-Eg) plot (Figure 6c) obtained from Tauc’s equation (Equation 2). The m value was calculated as 0.6 and this shows that Yb_2_O_3_ nanofibers have direct transition type [31, 32].


Equation 2 $$\text{F}({\text{R}}_{\infty })\text{h}\upsilon =\text{A}{\left(\text{h}\upsilon \text{-}{\text{E}}_{\text{g}}\right)}^{\text{m}}$$

where *hυ* is photon energy, *A* is energy-independent constant, *Eg* is optical band gap and *m* is exponent a value that determines bandgap types.

The optical band-gap of nanofibers was estimated as 3.32 eV by fitting the linear part of the graph of (F(R_∞_)hυ)^2^ vs photon energy given in Figure 6d.

**Figure 7:**
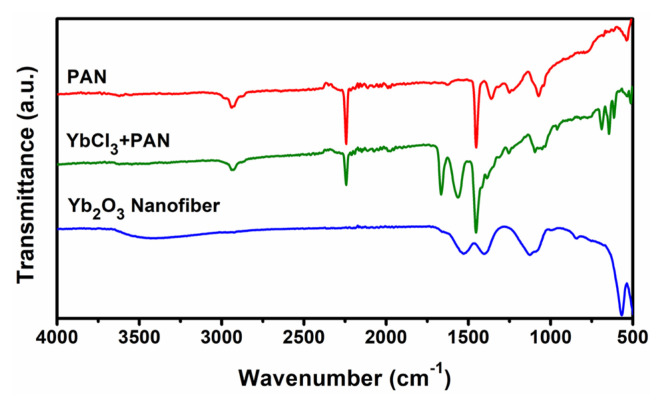
FTIR spectra results of Yb_2_O_3_, YbAc_3_+PAN nanofibers, and PAN.

FTIR spectroscopy was carried out to determine the presence of organic molecules in crystalized Yb_2_O_3_ nanofibers. FTIR results of crystalized nanofiber, polymer composite, and PAN are given comparatively in Figure 7. The FTIR results of PAN and composite nanofibers show that the characteristic peaks at 2935, 2245, 1448, 1360, 1242, and 1072 cm^−1^ are matched to the bond vibration of organic molecules belonging to PAN. The bands at 2935, 2245, and 1448 cm^−1^ were attributed to stretching of the C-H of the CH_2_, the C≡N of the acrylonitrile group, and the C=O of the carbonyl group, respectively. The peak centered at 1448 cm^−1^ occurred from CH_2_ tensile vibration, while the peak at 1360 cm^−1^ was ascribed to the C-H stretching of aliphatic CH groups. Furthermore, the C-H and C-O bond vibrations were observed at 1242 and 1072 cm^−1^, respectively [33, 34]. After the calcination process, these characteristic peaks of PAN disappeared due to the decomposition of organic molecules under the influence of heat treatment. Moreover, the peak at 568 cm^−1^ which is seen in the FTIR result of Yb_2_O_3_ nanofibers was assigned to bond vibration between Yb with O atoms.

## 4. Conclusion

Highly crystalline, pure, and homogeneous Yb_2_O_3_ nanofibers were fabricated by the electrospinning technique and presented to the literature for the first time with this study. The produced nanofibers were characterized by various methods such as XRD, SEM, TEM, elemental mapping, EDX, XPS, UV-Vis spectroscopy, and FTIR. As a result of the analyses, it was seen that the nanofibers consist of nanoparticles with 9 ± 2 nm, which have a pure and cubic crystal structure. Moreover, the chemical composition of the nanofibers was calculated as Yb_2.19_O_2.81_ by EDX. It was determined with optical measurements and calculations that the nanofibers have a direct band transition type and a band-gap value of 3.32 eV. The obtained characterization results show that the high crystalline Yb_2_O_3_ nanofibers can be used in many energy applications such as solar cells, hydrogen production, oxygen evolution-reduction reactions, and fuel cells. Moreover, it is predicted that produced nanofibers may contribute to an increase in application efficiencies because they have a larger contact surface area than other nanomaterials with different morphology used in these applications.

## Supporting Information

### Percentage of crystallinity

The percent crystallinity of nanofibers was calculated from the XRD pattern using the following equation.


$$X_{c}=\frac{A_{c}}{A_{c}+A_{a}}\times 100$$

where X_c_ is the degree of crystallinity, A_c_ and A_a_ are the areas of crystalline and amorphous peaks in the XRD pattern, respectively.

Figure S1. Modified Sherrer method graph of Yb_2_O_3_ porous nanofibers.

Figure S2. Williamson–Hall method graph of Yb_2_O_3_ porous nanofibers.

Figure S3. Diameter size distribution graph of YbAc_3_+PAN composite nanofibers.

Figure S4. Diameter size distribution graph of Yb_2_O_3_ nanofibers.
