# Supplementary figures and images for: Ytterbium oxide nanofibers: fabrication and characterization for energy applications
Source: Turk J Chem. 2022 Aug 15;46(5):1694–701. doi: 10.55730/1300-0527.3472 (PMC10390149; doi:10.55730/1300-0527.3472)

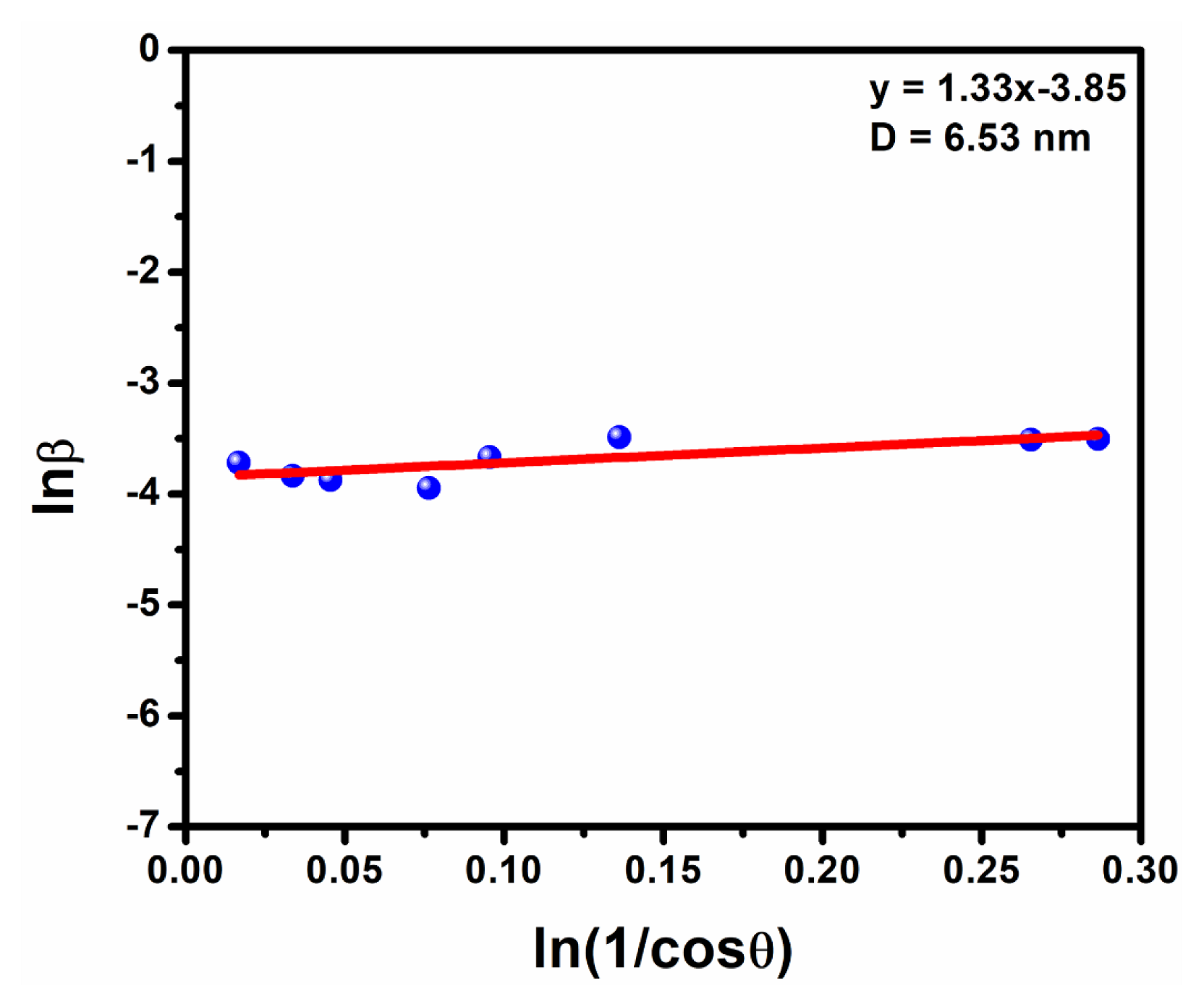

Supplement: Figure S1. — Modified Sherrer method graph of Yb2O3 porous nanofibers. [file turkjchem-46-5-1694s1.tif]

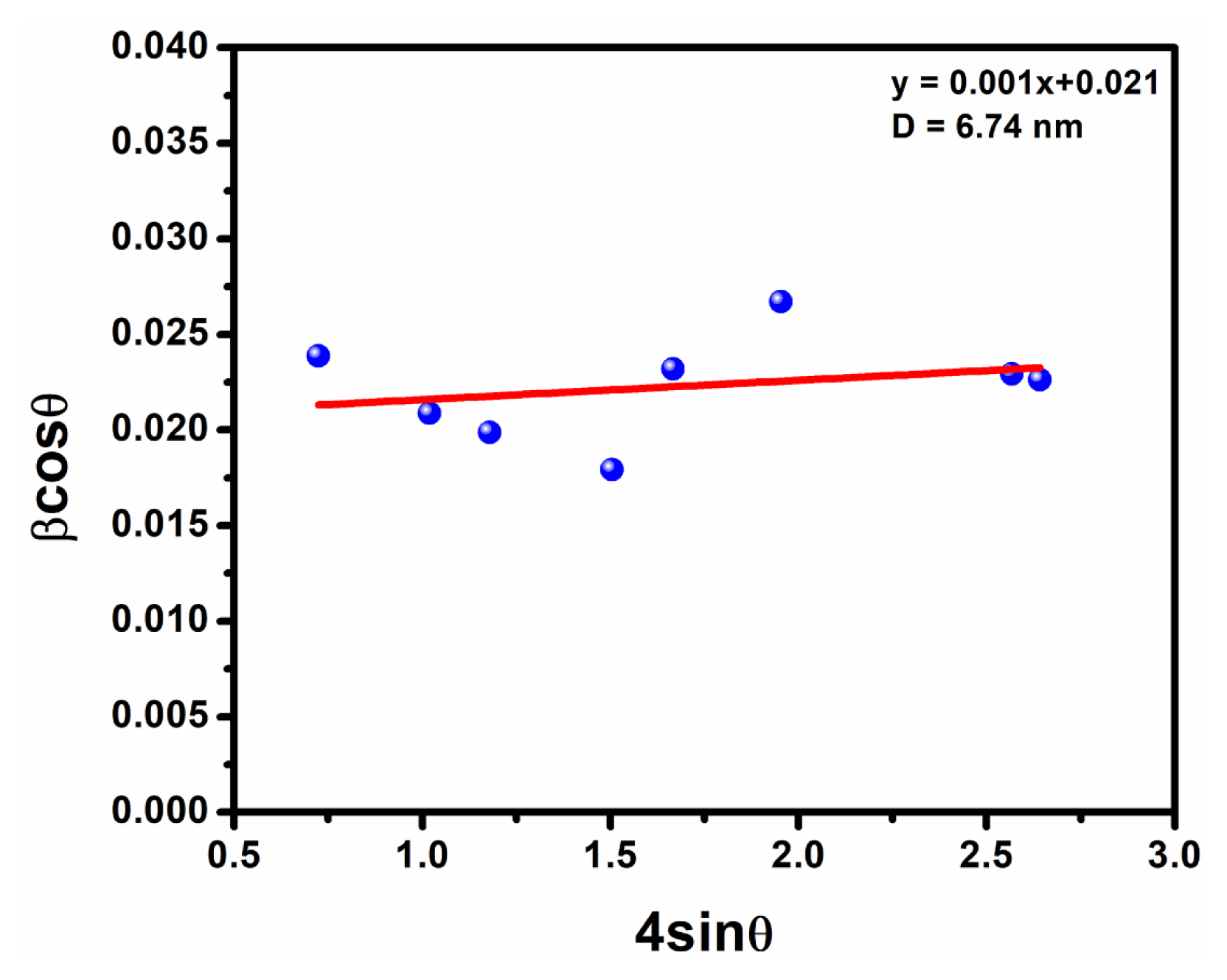

Supplement: Figure S2. — Williamson–Hall method graph of Yb2O3 porous nanofibers. [file turkjchem-46-5-1694s2.tif]

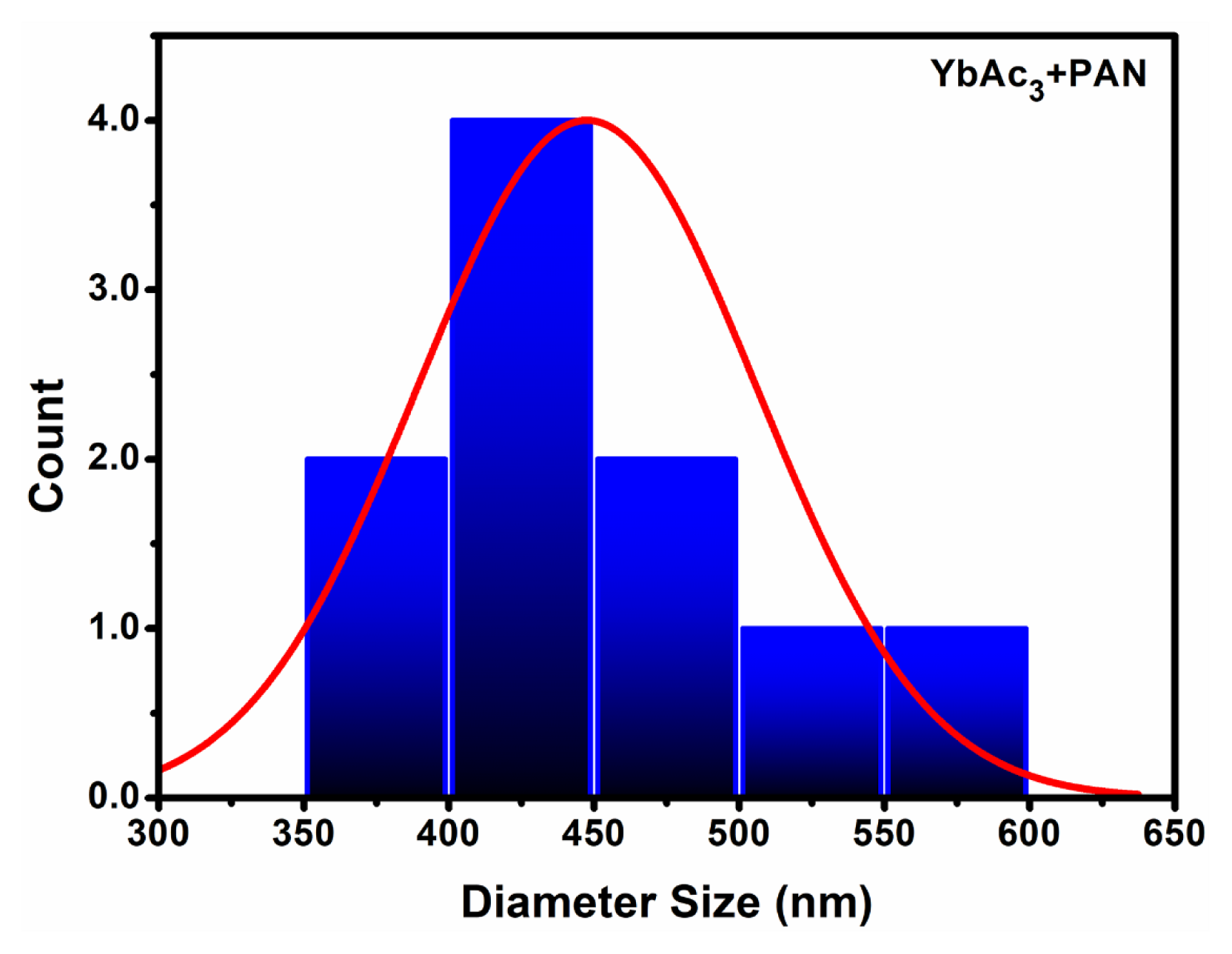

Supplement: Figure S3. — Diameter size distribution graph of YbAc3+PAN composite nanofibers. [file turkjchem-46-5-1694s3.tif]

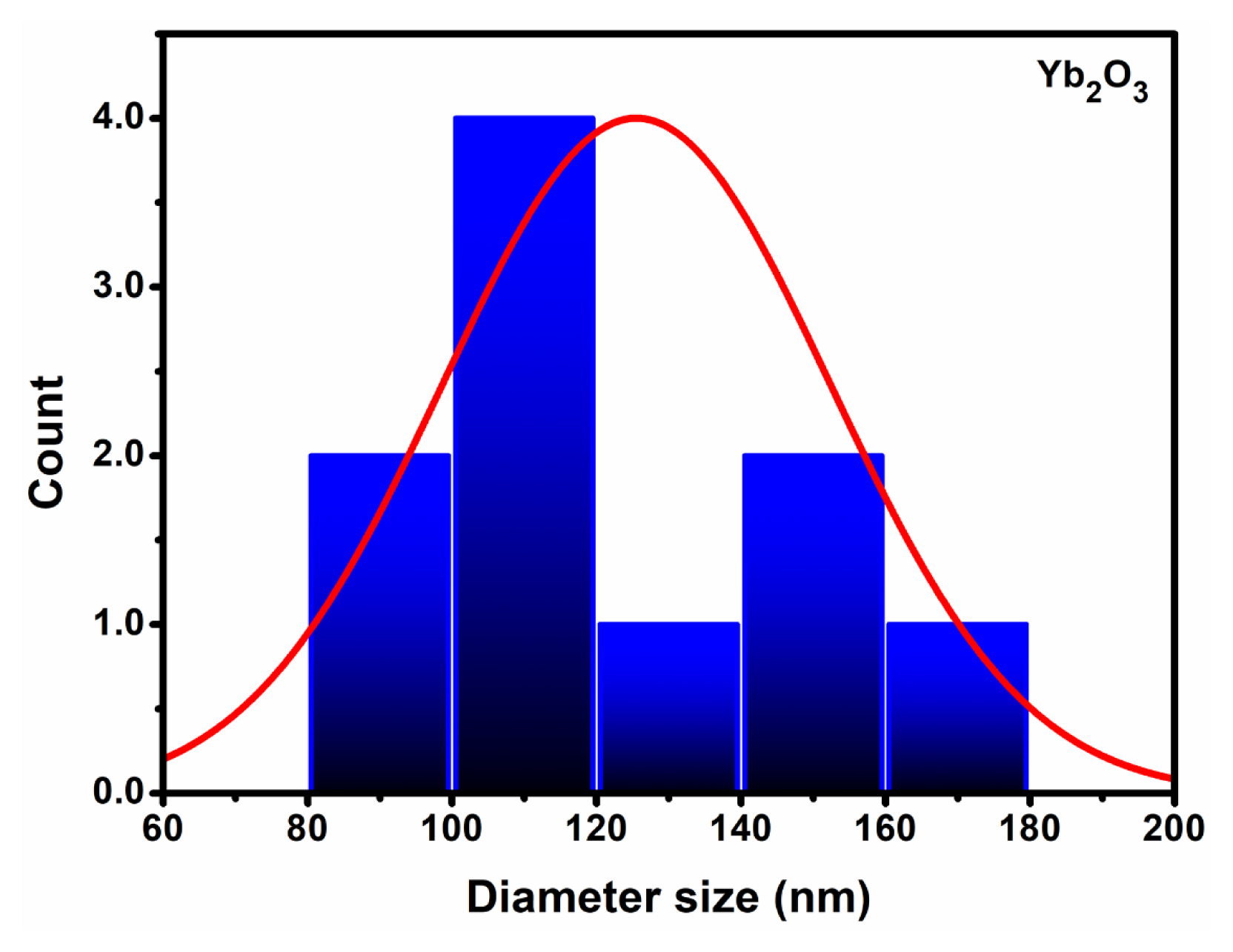

Supplement: Figure S4. — Diameter size distribution graph of Yb2O3 nanofibers. [file turkjchem-46-5-1694s4.tif]
